# Novel Scoring for Energy-Efficient Routing in Multi-Sensored Networks

**DOI:** 10.3390/s22041673

**Published:** 2022-02-21

**Authors:** Wooseong Kim, Muhammad Muneer Umar, Shafiullah Khan, Muhammad Altaf Khan

**Affiliations:** 1 Computer Engineering Department, Gachon University, Seongnam 13120, Korea; wooseong@gachon.ac.kr; 2 Institute of Computing, Kohat University of Science & Technology, Kohat 26000, Pakistan; muneer.umar@kust.edu.pk (M.M.U.); dr.altaf@kust.edu.pk (M.A.K.)

**Keywords:** IoT, WSN, routing, load balancing, energy efficiency

## Abstract

The seamless operation of inter-connected smart devices in Internet of Things (IoT) wireless sensor networks (WSNs) requires consistently available end-to-end routes. However, the sensor nodes that rely on a very limited power source tend to cause disconnection in multi-hop routes due to power shortages in the WSNs, which eventually results in the inefficiency of the overall IoT network. In addition, the density of the available sensor nodes affects the existence of feasible routes and the level of path multiplicity in the WSNs. Therefore, an efficient routing mechanism is expected to extend the lifetime of the WSNs by adaptively selecting the best routes for the data transfer between interconnected IoT devices. In this work, we propose a novel routing mechanism to balance the energy consumption among all the nodes and elongate the WSN lifetime, which introduces a score value assigned to each node along a path as the combination of evaluation metrics. Specifically, the scoring scheme considers the information of the node density at a certain area and the node energy levels in order to represent the importance of individual nodes in the routes. Furthermore, our routing mechanism allows for incorporating non-cooperative nodes. The simulation results show that the proposed work gives comparatively better results than some other experimented protocols.

## 1. Introduction

The Internet of Things (IoT) [1] was recently made practical with the adoption of some state-of-the-art technologies, such as wireless sensor networks [2] and intelligent sensing [3]. The applications of IoT include health care, inventory tracking, smart grid networks, security systems, and maintainable transportation. The interconnected smart objects with embedded sensors in the IoT network cooperate and coordinate with one another to send the collected data to a gateway sink. For IoT-based applications, such as industrial control, environmental sensing, smart homes, and logistics management, the wireless sensor network (WSN) is an essential part of the infrastructure [1]. The WSN can be represented as a graph of multiple interconnected sensor nodes, where each node senses some data from the environment and transfers them to an ultimate station. The infrastructure of IoT-based WSNs can be autonomously organized without any complicated time-consuming installation and configuration compared to typical wired networks for a variety of purposes [2].

In WSNs, nodes operate with limited powered batteries and cannot be recharged or replaced in a short period since the sensor nodes are typically unattended. The various applications of WSNs in IoT environments suffer from this limitation. Accordingly, most of the previous research works have focused on the extension of the lifetime of the nodes while achieving peak throughput [4]. In WSNs, data transmission is done through the nodes cooperating with one another since most of the nodes may not have a direct connection to a sink node; the nodes use other nodes as relays for transferring their sensed data, which is known as multi-hop communication. For multi-hop communication in a WSN, a node probably has multiple options to select a path towards a destination. Many researchers have proposed various routing schemes considering routing parameters such as the nodes’ energy levels, transmission rate, security, and so forth [5]. 

In IoT-based WSNs, the energy consumption of sensors is a major concern. Therefore, the effects on energy consumption have been investigated in most of the legacy routing protocols. Moreover, many routing schemes are designed with particular focus on the energy preservation and elongation of the network lifetime [6]. The goal of energy management is to ensure that the sensors perform for longer periods of time and all the sensors consume their energies equally [7]. Different techniques have been developed to balance the load and energy consumption among the nodes [8]. However, it is unavoidable that some nodes in the network do not cooperate for the sake of saving their energies. Such non-cooperative nodes behave selfishly either temporarily or forever. These selfish nodes severely degrade the overall network performance. In most of the legacy mechanisms, therefore, the selfish nodes are either isolated or blocked [9].

For energy-efficient routing, various mechanisms have been proposed [10]. Sleep scheduling approaches are of recent special interest to the scientific community, as they allow for some nodes to be idle for a particular period of time [11]. In most sleep scheduling mechanisms, the density of the nodes at various locations in a network is considered. In [12], the authors proposed the usage of some approaches combined in a genetic algorithm to formulate a discrete particle swarm optimization algorithm. The main objective of such mechanisms is to preserve the idle nodes for future operations that are redundantly deployed in the network. Moreover, it was shown that the sleeping nodes cause no negative impact on the overall performance of the network. Thus, the sleep scheduling mechanisms are very efficient for energy optimization in WSNs. However, these mechanisms purely rely on the density of nodes and become ineffective when there are no redundant nodes in the network. Moreover, some nodes that die over time also reduce the redundancy and degrade the impact of the sleeping scheduling.

In some proposed mechanisms [13], it is assumed that the nodes cooperate with each other while conducting a common routing protocol. However, in ad hoc and IoT networks, the smartness of nodes is very common. Therefore, this aspect must be properly addressed in such types of networks while designing an energy-efficient scheme. Many schemes focus on the individual contribution of each node towards energy efficiency by adapting a routing protocol [13], sleep behavior [11], coordination mechanism, data aggregation procedure [7], hop division [9], and cluster divisions [14], and so forth. The nodes may intelligently coordinate with each other considering each node’s status. The nature of the deployment of the nodes also has a significant impact on the performance and lifetime of the networks. The energy efficiency techniques should adequately utilize the density or redundancy of the nodes in such types of networks [15]. There should be sufficient space in the mechanism to consider as many parameters as possible for designing an energy-efficient routing in a WSN-based IoT network. The parameters can be the selfishness of the node, neighborhood, connectivity through hop levels, density of the nodes, redundancy of nodes, energies, distances, and surrogate values such as points, score, or credit values for nodes, and so forth.

In this work, we propose the node status and score-based route optimization protocol (NSSROP), where each node keeps some additional data to balance the routing load among all the nodes. In an IoT setup, a sensor node may have the shortest available route towards a sink. However, it should wisely choose a route that balances the load and elongates the life of the entire network. Some nodes may be placed at a location where they may get a higher rate of relaying requests compared to other nodes. This situation can highly degrade the network performance by unbalancing the load among the nodes. For this purpose, each node calculates some values for itself that are referred to as scores. Unlike other typical routing protocols, the proposed mechanism addresses various parameters associated with the routing and energy optimization in the network. These parameters are used for the calculation of the scores. During the selection of a route by the source node towards the central control, each forwarding node builds the route by considering these scores of the relay nodes. Modified route request (RREQ) and route reply (RREP) packets are used to exchange the variations in these scores. 

The remainder of this paper is ordered as follows: In Section 2, related works are described. In Section 3, the preliminary formations are described and the whole mechanism is explained. In Section 4, the simulation results are discussed. Lastly, Section 5 includes the conclusion and future work.

## 2. Related Work 

The routing in IoT sensor-based networks is one of the most remarkable research areas in communication networks. There are lots of research articles related to this field. There are various parameters in the network that can be used to optimize the routing, for example, optimization with load balancing (traffic load distribution), as discussed in [16]. 

A proactive tree-based routing protocol, the routing protocol for low-power and lossy networks (RPL), is defined by RoLL [17]. RPL is a standard protocol that operates on an IPv6-based IoT network. It brought an opportunity to develop WSNs on a very large scale. Routing and message control are the RPL’s most important mechanisms for establishing and maintaining an effective and stable network. Despite its standing as the standard routing protocol for IoT networks, RPL has had various flaws since its inception, and other approaches have emerged to address them [18]. Among these, routing loops are critical.

Most of the recent studies that have aimed at energy efficiency and load balancing in WSNs and WSN-based IoT networks preferred the cluster-based approach. In [14], the authors proposed the integration of the bat algorithm and low-energy adaptive clustering hierarchy (LEACH) for the efficient cluster head selection to reduce energy consumption and balance load among the network nodes. The nodes are also bound to follow a schedule for the transmission of their data packets. This mechanism primarily focuses on the cluster head selection by considering the nodes’ energy levels. Each cluster head is bound to have a particular number of connected cluster members. However, unlike this mechanism, the distribution of nodes in a network can be random, which makes it difficult to specify the number of nodes for each cluster. 

Turgut and Altan [19] introduced a fully distributed energy-aware multi-level (FDEAM) routing and clustering mechanism for WSN-based IoT networks. The two-level and multi-level inter-cluster transmission methods are represented in this work. In the second level, the communication and transmission strength are determined by considering the distance between the nodes and the base station (BS). The clusters are statically distributed over the entire network. However, the option for re-clustering the network is also defined. Self-arranged nodes elect the limits for clustering, and cluster heads are selected by executing the FDEAM method. However, this method is inappropriate for non-uniform node distribution and has the shortcoming of being reliant on a dominant source. 

The authors of [20] presented an energy-efficient architecture of a self-sustaining WSN based on an energy-collecting BS and a mobile charger considering the cost of deployment. They conducted extensive simulations and demonstrated the efficacy of their proposed strategy by showing that it maximizes the expected network lifetime while minimizing deployment costs. The main idea is focused on the usage of mobile chargers and the energy-harvesting BS. However, the work did not primarily deal with energy-efficient routing. 

The position responsive routing protocol (PRRP) was proposed in [21]. The main objective of this proposed work was to minimize energy consumption by incorporating the global positioning system (GPS) into the nodes. The network is divided into equally sized grids with a static or dynamically distributed number of nodes. The nodes can adjust their transmission power by using GPSs while communicating with each other.

To balance energy consumption within each cluster, Wang et al. [22] suggested uneven cluster generation and distributed cluster head rotation based on residual energy and relative location. The authors also designed a routing path updating system to prevent node energy depletion. The selection of the cluster head is based on the level of residual energy of the nodes. The routing paths are dynamic and also associated with the nodes’ energy levels. 

An energy-efficient regional source routing protocol was proposed in [23], which balances the network’s energy usage by dynamically picking cluster heads with the most remaining energy among the WSN nodes. Furthermore, the ant colony algorithm based on distance is employed to determine the global ideal transmission path for each node, which reduces data transmission distance and energy consumption. The experiment results show that the proposed approach outperformed the compared approaches in terms of network lifetime and throughput.

The authors of [24] proposed open vehicle routing (OVR) based on fundamental WSNs parameters, in which a data collection protocol called EAL improves the energy efficiency by balancing the lifetime of the network nodes while considering latency. 

Han et al. [25] proposed a cross-layer routing protocol for optimizing the routing in geographic node disjoint multi-paths. The routing layer performs according to the underlying energy demand of the network nodes while the physical layer adjusts the transmission power according to the energy levels. The authors also applied sleep and awake states for energy saving. In [26], the higher level of traffic generated by several source nodes in an IoT environment was considered. Three factors are used to determine optimal routes by taking the next hop nodes. These factors include (1) the signal to interference and noise ratio, and (2) the survivability factor and congestion level of the preferred forwarding node. 

The Path Operator Calculus Centrality (POCC) routing protocol was proposed in [27]. POCC is used to determine the nodes’ centrality scores, which are further used for path determination. The approximation of the centrality score uses the operator calculus method based on the topology of the network. The authors argue that this technique provides optimal paths towards the BS. The article [28] proposed a directional transmission-based energy-aware routing protocol (PDORP) to find energy-efficient routes. The DSR protocol is used as a base protocol in this mechanism. Moreover, a hybrid of bacterial foraging optimization and a genetic algorithm is used to efficiently collect node information. The authors presented comparatively better results for energy consumption, bit error rate, delays, and throughput from their experiments. The objective of this work was to attain a better quality of service and extend the network life. The predicted remaining delivery (PRD) protocol, based on the path weighting technique, was proposed in [29]. PRD considers the fundamental parameters, such as route quality, residual energy, end-to-end delays, and inter-node distance for designing the weightage system. 

A well-known approach for selfish node management was introduced in the watchdog and pathrater method [30]. In this work, the watchdog detects the non-cooperative behavior of the nodes and the pathrater blocks the selfish nodes from being part of the routes. The presence of non-malicious selfishness is potentially higher in unlicensed entities in an IoT infrastructure. Therefore, it is critical to block the unwanted nodes in such a network.

Many research works have described mechanisms for determining and utilizing nodes’ individual importance in a network. Sun et al. [31] proposed an important assessment mechanism for a particular node with respect to the energy field. They determined key nodes based on the average length and density of nodes for the stability of a network. For this, the authors used graph theory for the properties and correlation of the nodes with the energy field. In another work [15], an evaluation index was introduced based on the topology of the network, which eventually determines the nodes’ locations within a network. Additionally, supernodes are designated to manage multiple key nodes within the network.

In a mechanism proposed in [32], the selection of relay nodes is made by a concept of “equivalent nodes” based on a proposed energy consumption model. The network life can be lengthened by applying a probabilistic dissemination algorithm among those relay nodes. 

Some fuzzy logic-related articles have also been proposed to improve the energy efficiency in WSNs. Sheriba et al. [33] proposed a fuzzy logic and black widow optimization clustering protocol. However, the black widow optimization’s ideal performance is modest. Later, the authors proposed a strategy for designing the optimal interval type 2 fuzzy logic by involving the evolutionary algorithms [34]. This solution technique is suitable for WSNs with limited energy since it helps to extend the network’s lifespan. In reference [35], a trust-aware energy-saving stable clustering algorithm based on the fuzzy type-2 algorithm was devised to solve the constraint of the shortening lives of the cluster heads in clustering algorithms.

Various studies have proposed game-theoretic approaches for the establishment of a tradeoff between the desired signal-to-noise ratio (SNR) and energy consumption [36,37]. These approaches focus on optimal route selection while considering communication quality. The game-theoretic approach is effective in the sense of getting a payoff for individual nodes. However, the entire network’s performance cannot be optimized by these approaches. Moreover, the nodes are self-focused in such approaches, and these do not give any length to the network life. The node selection mechanisms such as those in [27,38] were also proposed for choosing the best nodes among others for energy optimal efficiency in the network.

Various nodes and network scoring mechanisms have been proposed by many articles mainly focused on energy efficiency, node behavior, and security. The GoNe scheme, proposed in [39], was designed for enforcing data security and privacy in WSNs. Nodes are given some scores based on their reputation in the network. These reputation scores are managed by CHs, which are later used to manipulate the behavior of nodes. In another score-based load management scheme [40], the authors proposed a mechanism to compress the data through CHs to reduce the load on the nodes with low scores. The best nodes are chosen based on their remaining energy and distance from the BS. The CHs use compressive sensing to compress data and then forward information towards the sink through the best nodes. The authors claim that in this way, the load is balanced among all the nodes. The SBRR protocol [41] considers many factors to score paths for nodes. The parameters are the hop count, the remaining energy of nodes, link quality, and the buffer sizes on the nodes. All the parameters are integrated to form the path score. The main focus of the work was to reduce the pack loss in the transmission. Still, there is space for load balancing and energy efficiency in the work.

## 3. The Proposed Mechanism

Unlike RPL and other existing routing protocols, the proposed mechanism addresses various parameters associated with routing and energy optimization in the network. A node scoring mechanism is introduced based on the nodes’ existence in the network. The neighborhood of a node is further classified as closed or identical neighbors. Some of the procedures in this study were influenced by our previous work on reward-based mechanisms (RwBMs) [9]. The RwBM is a game-based approach that uses the Rubinstein bargaining game for the management of virtual currencies which are referred to as scores. Our previous work deals with the management of selfish nodes in WSNs using the RwBM mechanism. In this study, we proposed a novel scoring scheme to select the best relay nodes while choosing a path. Herein, the key algorithm for the score manipulation and calculation, which involves entirely different procedures, is distinguishable from the RwBM.

This section is divided into two major subsections. In Section 3.1, the preliminaries are discussed, and in Section 3.2, the entire mechanism is explained in detail.

### 3.1. Preliminary Formation for the Proposed Work

This section presents the basic details of the main mechanism of the NSSROP. Each node in the network contains the following information:

#### 3.1.1. Hop Level

The networks are divided into a hierarchical format of interconnected nodes. The intermediate nodes with a direct connection to a sink are denoted as having a hop level of one, while the nodes at the end boundaries have the maximum level values. Each node keeps its own hop level to determine its distance from the sink. The hop level can be used to define the number of nodes in a route towards the sink. Equation (1) shows the hop level for a node within a network of ***n*** nodes, as follows:

(1) $$1\leq \;HL_{i}\leq n\;\;$$

#### 3.1.2. Neighbors

In multi-hop communication, the presence of neighboring nodes plays a vital role. A higher number of neighbors leads to a higher availability of routes towards the sink. Each node in the network keeps a list of all possible nodes that are in the transmission range. The neighbors can be classified as upward, downward, or sibling nodes. Upward nodes are neighbor nodes one hop level up. Downward nodes are the neighbors with a lower hop level, while the siblings are the nodes with the same hop level. A node with *HL* equal to *HL^max^* indicates that this node is at the very bottom of the network. Such nodes do not have downward nodes so they only transmit data through forwarding or sibling nodes.

#### 3.1.3. Closed Neighbors

Among the neighboring nodes, some nodes are relatively placed closer than other neighbors. Such nodes can be considered as closed neighbors. However, it is more appropriate to consider this distance based on the received signal strength indicator (RSSI) mechanism than the physical distance. An RSSI-based distance value is calculated by Equation (2) and is used to determine the set of neighbor nodes [42].

(2) $$DST_{i,j}=P_{i,j}^{-1}\left(d\right),\;\;\;P_{i,j}\left(d\right)=\frac{p_{i\;}G_{i\;}G_{j\;}\Lambda^{2}}{{\left(4\pi \right)}^{2}\;d^{2}}$$

where $p_{i\;}$denotes the transmission power, and$\;G_{i}$ and $G_{j}\;$denote the antenna gains of nodes *I* and *j*, respectively. Nodes *i* and *j* are the transmitter and receiver, respectively. Λ indicates the wavelength (meter) of the transmission signal. $p_{i,j\;}$is the receiving power at the node *j* when the inter-node distance is $d_{\;}$. In a constrained situation where the sensor nodes are deployed in a controlled environment, the Pythagoras two-dimensional distance formula can also be used for creating the set of CNs. Moreover, if nodes are deployed in irregular, unaligned, or not plane areas, then the same can be converted into the three-dimension distance formula. 

Each node keeps a separate set of closed neighbors (CNs) with their estimated location and energy information. If a node has a frequent number of CNs, then it means that the node has less opportunity to become a forwarder of other nodes. Nodes with a fewer number of CNs are vital and can perform more than others. The set of CNs can be calculated as follows:

(3) $$CN_{i}=\left\{j:DST_{i,j}\leq \;DST^{tr}\right\}$$

*CN_i_* are all the nodes that are located within a distance value *DST^tr^* with a specified RSS threshold from node *i*.

In Algorithm 1, the distance of the inputted node is compared with all the nodes from 1 to *n*. In each iteration, the computed RSSI-based distance is checked for whether it is less than a predefined threshold distance for the CNs. Stack memory is used to store all the nodes that are at a concerning distance with the inputted node, that is, equal or less than the threshold distance. In case no CN of a node exists, this function returns 0. 

**Algorithm 1.** Calculation of CNs for a given node (node_ID).

$CALCULATE\_CN\left(node\;ID\right)\;1.\;\mathrm{for}\;i=1\;\mathrm{to}\;n\;2.\;\mathrm{StackCNs}.\mathrm{Top}=0\;3.\;\mathrm{if}\;(\mathrm{ID}\neq i)\;//\mathrm{case}\;\mathrm{of}\;a\;\sin\mathrm{gle}\;\mathrm{node}\;4.\;\;DST_{i,ID}=P_{i,ID}\left(d\right)=\frac{p_{i\;}G_{i\;}G_{ID\;}\Lambda^{2}}{{\left(4\pi \right)}^{2}\;d^{2}}\;\;\;\;\;\;\;\;\;\;\;\;\;\;\;\;\;\;\;\;\;\;\;\;\;\;5.\;\;\mathrm{if}\;0<DST_{i,ID}<DST^{tr}\;6.\;\;\;Push\mathrm{Ii}\;\mathrm{to}\;\mathrm{StackCNs}.\mathrm{Top}\;7.\;\;\;\mathrm{StackCNs}.\mathrm{Top}=\mathrm{StackCNs}.\mathrm{Top}+1\;8.\;\;\mathrm{end}\;\mathrm{if}\;\;9.\;\mathrm{end}\;\mathrm{if}\;\;10.\;\mathrm{end}\;\mathrm{for}\;\;11.\;\mathrm{Return}\;\mathrm{StackCNs}\;$



$DST_{tr}$ is a threshold value used to limit the succeeding nodes to being *CNs* with a specific node. This value can be wisely defined by the consideration of the total number of nodes, nodes’ placements, and the transmission range of the nodes. If the number of nodes is higher in a particular field, then we can assume that the nodes are more densely located. Similarly, the nodes with lower transmission power will make most of the nodes directly unreachable to each other. The value of $DST^{tr}$ should be less than the maximum RSSI value in the network; otherwise, the parameters of the *CNs* will have no or an erroneous impact on the mechanism. Moreover, if we use a very small $DST_{tr}$ value, then it will return none or a smaller number of *CNs.* The proposed work uses the *CNs* for the network optimization; therefore, the reasonable number of *CNs* has a greater impact on the overall performance of our proposed work. In our work, for the experiments, we kept this value as half of the maximum possible RSSIs. 

#### 3.1.4. Identical CNs

It is probable that at some particular locations in the sensor field, some nodes reside at a very near distance to some other nodes. Such closely located sensor nodes generally sense data in parallel and get RREQs from the same source nodes. Two or more closely deployed nodes with the same connections to other nodes can be denoted as identical to each other. Such nodes will have relatively similar sensed data and similar RREQ from other nodes. It is feasible to utilize such nodes by assigning them more load compared to other nodes. For closely related nodes, an appropriate distance must be configured. A higher distance leads to a larger number of identical nodes, which may cause a negative impact. Contrarily, a lower value reduces the sets of identical nodes, making the proposed work ineffective. In our experiments, we assumed that the nodes with a distance of a quarter of the maximum transmission range of the nodes were identical CNs. 

**Figure 1:**
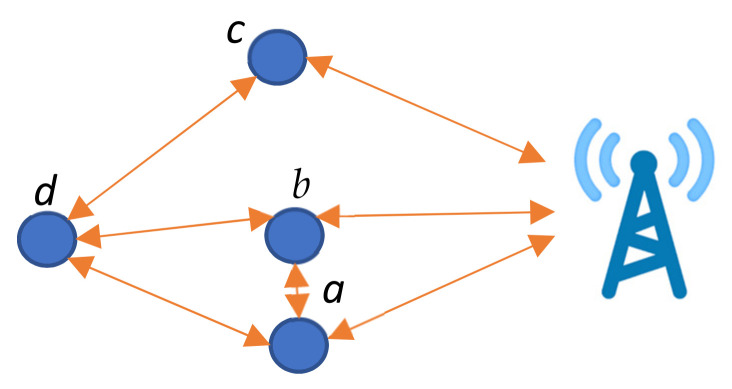
Identical CNs (*a* and *b*).

In Figure 1, nodes *a* and *b* are CNs of each other and also have similar connections to other nodes, while node *c* is alone and has a similar hop level. In case one of the identical nodes exhausts its energy completely, the effect is limited compared to a stand-alone node. Therefore, it is wise to utilize nodes *a* and *b* more frequently than node *c*.

#### 3.1.5. Energies of Neighbors

The main concern is energy optimization in the IoT sensors. Therefore, each node keeps its own as well as its neighbors’ energy information. The energy of node *i* can be denoted as *E_i_*. The values of *E_i_* can be determined as follows:

(4) $$0\leq E_{i}\leq E^{max}$$

The nodes with energy levels equal to 0 are considered dead nodes. Such nodes are automatically omitted from the network. The features of the dead nodes cannot be used in the formation of scores for routing. Each node keeps track of its neighbors and deletes the dead nodes from the neighbors’ lists.

#### 3.1.6. Scores

In the network, each node governs a routing table that is used for sending its data. For each source to its destination, a sequence of nodes is kept in this table. The routing table keeps the sequence of nodes in each possible route for a destination. A set, *R,* can be defined to show all the possible routes for node *i* via the presence of intermediate nodes. Each route can be denoted by r with a sequence of nodes.

(5) $$R_{i}=\left\{r_{1},r_{2},r_{3},\ldots \;,r_{j}\right\}$$

The nodes present in the routes can be distinguished by their importance using a scoring mechanism. The routes can be determined by evaluating the scores of the nodes. 

The efficiency of a route can be calculated by considering the nodes’ density, that is, the number of CNs and their energies at each hop level towards the sink. Each transmission of the data packets from a source consumes the energies of all involved nodes. The deduction of energies induces a variation in the scores of nodes. The score of each node *i* can be calculated by its energy level, the sum of the energies of its CNs, and their size *m* at time *t*. The score of node *i* at time *t* can be derived by Equation (6) as follows:

(6) $$\lambda_{i}^{t}=\frac{E_{i}^{t}}{E^{max}+\sum_{j=1}^{m}E_{CNj}^{t}}\times \left(m+1\right)$$

The high level of energy of node *i* and/or a higher number of CNs leads to a higher value of *λ.* Additionally, CNs’ energies also influence this value. The higher-level CN energies reduce the value of *λ*. For an exceptional case in which a node does not have any connected or dead CNs, *λ* is calculated by dividing the energy of node *i* by the maximum level of energy, as follows:

(7) $$\lambda_{i}^{t}=\frac{E_{i}^{t}}{E^{max}}$$

Since each route may have multiple nodes, the *λ* of relay nodes is considered at each level. The main aim of considering the node density is to reduce the load on single or scarce nodes. Once the system starts putting the load on the densely located nodes, it is obvious that at some following stages, some of the nodes will exhaust their energies and will ultimately no longer operate. The load is finally diverted to other nodes.

Algorithm 2 is used to calculate the λ values for the nodes. This algorithm uses a subroutine, CALCULATE_CN(ID), to get the list of all the CNs for a specific node. The number of all CNs is retrieved and then according to Equation (6), these CNs are processed using a simple loop.

**Algorithm 2.** Calculation of *λ* for each node in the network.

$LAMBDA\left(node\;ID\right)\;1.\;\mathrm{ID}.\mathrm{CNs}={\mathrm{CALCULATE}}_{CN\left(ID\right)}\;2.\;\;\lambda_{D}=\mathrm{MAXEnergy}\;\;3.\;\mathrm{TotalCNs}=\mathrm{Size}\;\left(\mathrm{ID}.\mathrm{CNs}\right)\;//0\;\mathrm{is}\;\mathrm{assigned}\;\mathrm{if}\;\mathrm{no}\;\mathrm{CN}\;\mathrm{exits}\;4.\;\mathrm{if}\;\left(\mathrm{TotalCNs}\neq 0\right)\;5.\;\mathrm{for}\;i=1\;\mathrm{to}\;\mathrm{TotalCNs}\;6.\;\;\mathrm{TempCN}=\mathrm{ID}.\mathrm{CNs}\left(i\right)\;7.\;\;\lambda_{D}=\lambda_{D}+\mathrm{TemptCN}.\mathrm{Energy}\;8.\;\;\;\lambda_{N}=\left(\mathrm{TotalCNs}+1\right)*\mathrm{ID}.\mathrm{Energy}\;9.\;\;\;\lambda =\lambda_{N}/\lambda_{D}\;10.\;\mathrm{Return}\;\lambda$



#### 3.1.7. The Reputation of Hop Level Neighbors 

Some nodes may not cooperate due to their selfish behavior. Such nodes are enlisted during the data transmission by a source node. If a node does not reply to a route request, then it is considered a selfish node. The selfish nodes are not served by other relay nodes for data transfer requests. In detail, if a node continues non-cooperative behavior for a long period of time T, then it is black-listed. In addition, the blacklisted nodes are not requested for relaying services. A counter Cr is used when the source node declares a node as a blacklisted one after being selfish. A list of selfish and blacklisted nodes is broadcasted among the neighbors so that they might be contacted adaptively by all neighbor nodes. The states of selfish and blacklisted nodes can revert to normal after a specified period of time.

Due to the existence of selfish nodes in the WSN, unfavorable situations may exist. For instance, it is possible that a node may not be able to communicate or transfer its data to the sink due to the presence of one or more selfish nodes along the route. In the worst case, a node can meet relay nodes that are all selfish. In such cases, these nodes cannot communicate with their destinations [9]. Such nodes typically attempt to retransmit data repeatedly during a particular period of time. In this study, we assumed that multiple alternate paths for transmissions were available in order to cope with such network partition by selfish nodes. Due to the fact that node intelligence encourages selfish behavior, a node must be able to select only the reliable ones among multiple relays to prevent excessive packet drops.

#### 3.1.8. Routing Information Formats

**Figure 2:**
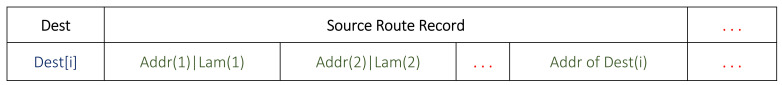
Modified routing table format.

The format of the modified DSR route table is given in Figure 2. This table demonstrates the format of a single entry for a destination node *i*. In the routing cache, an additional 2-byte Lambda (*λ*) value is concatenated with each address. Nodes can easily update these according to their own knowledge. Each node transmits its own λ field through the routing and topology control messages.

**Figure 3:**
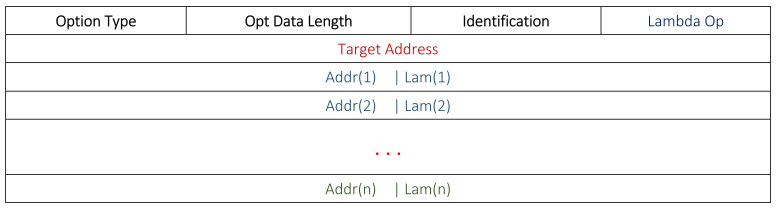
Modified DSR RREQ format.

**Figure 4:**
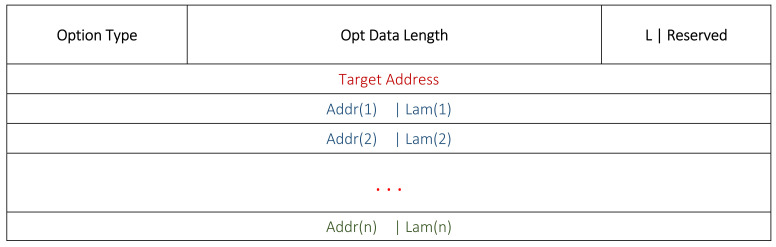
Modified DSR RREP format.

Figure 3 and Figure 4 show the RREQ and RREP formats in this work. An additional 1-byte field for the *Lambda Option* is added in the header. The presence of this field specifies the addition of ***λ*** values with each address. Usually, the *Lambda Option* is kept as null in the RREQ to avoid any additional bandwidth and energy. In the RREP, we used the modified addresses fields. Accordingly, each relay node adds its own address with its ***λ*** score for the RREP. 

**Figure 5:**
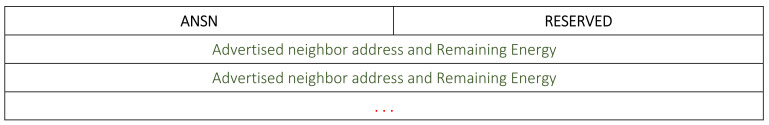
Information exchange through modified OLSR topology control message.

Most of the time, the nodes estimate their neighbors’ locations and energies by analyzing the sequence of the involved nodes in the flow of data transmission from a source towards a sink. However, sometimes nodes may require an update for these values after a specified period of time. OLSR topology control messages are used to get the neighbors’ locations and their energies. For this, we modified the OLSR topology control message as shown in Figure 5, where each address is combined with the nodes’ energies for the sake of information sharing. 

An unsigned integer 2 bytes in size is used to represent the value of ʎ. According to Equation (6), the value of Lambda must be a decimal ranging from 0 to the maximum number of nodes. The possible value range for 2 bytes is from 0 to 6.5535. To adjust the decimal values into an integer of 2 bytes, the value of ʎ is rounded to 4 decimal points and then multiplied by 1000. After applying this procedure, the mechanism can use the maximum value of ʎ up to 6.5535. However, there is the possibility that this value will be near the total number of nodes in the network. When we used an appropriate value for calculating the set of CNs, there was a much lower chance of this value being greater than 2 in most of the experimental cases. For example, a sample of calculated values can be seen in the next section of Simulation Results. A value greater than normal will have the same impact no matter how much greater it is. So, during the process, we assumed that any value of ʎ greater than 6.5535 must be considered 6.5535.

### 3.2. Proposed Mechanism

**Figure 6:**
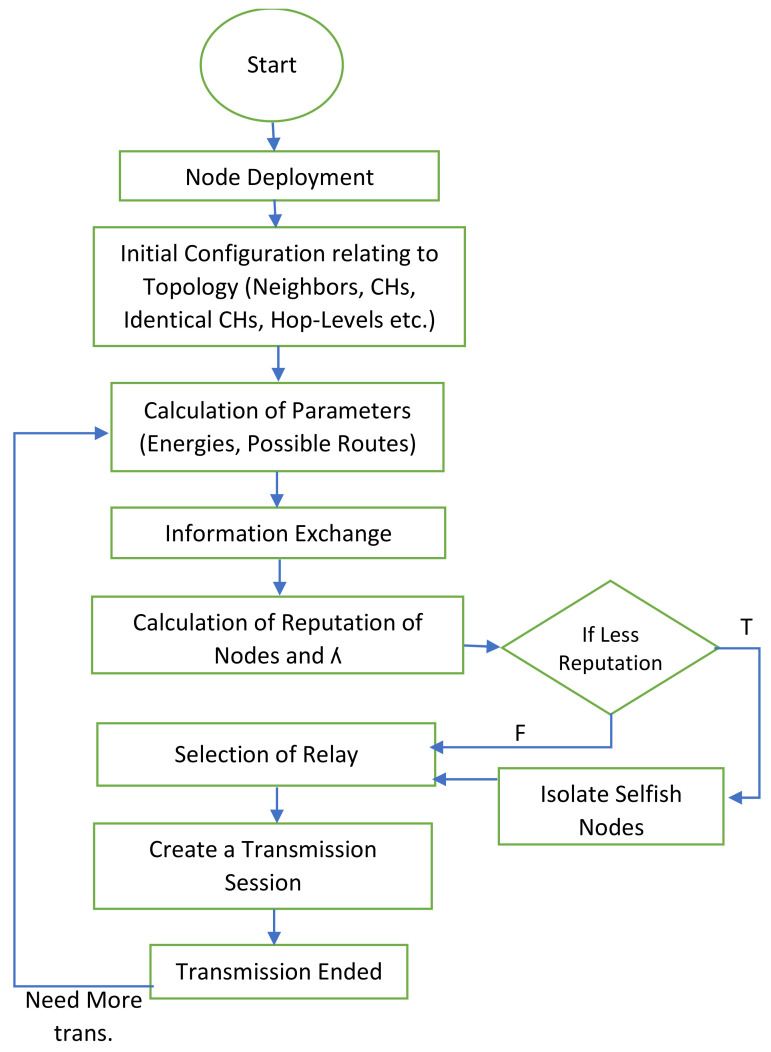
Flowchart of the proposed mechanism.

The flowchart for the entire process of our algorithm is depicted in Figure 6. Our proposed mechanism is divided into sub-parts of the initial configuration, the selection of relay nodes, handling the selfish nodes, and the information exchange.

#### 3.2.1. Initial Configuration

**Figure 7:**
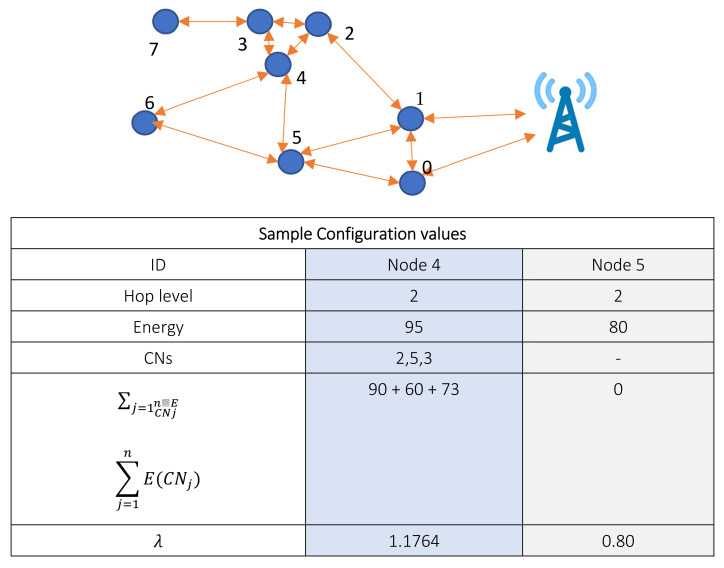
Initial self-configuration of nodes.

After the deployment of the network, initially, each node broadcasts control packets to determine its location, neighbors, and CNs and their energy levels. After learning the values of other connected nodes, each node builds its routing table with routes towards a sink through the relay nodes. Since the main concern is to select the optimal route towards the sink, each node *i* calculates its *λ* at time *t*. This value is then shared with the neighbors. The initial configuration can be seen in Figure 7, where two sample nodes are shown with their recorded parameter values. 

#### 3.2.2. Selection of Relay Nodes

**Figure 8:**
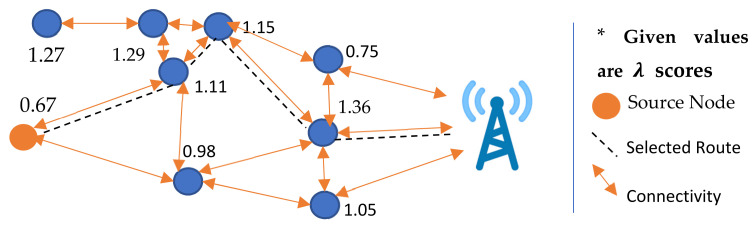
Selection of relay nodes.

While selecting a route, the source node sends RREQs to an upper-hop node that has the highest λ value. Upon receiving the RREQ, the relay node then further requests its ascendant node, which has a higher λ value. This process is repeated by all forwarders until the intermediate nodes with a hop level of one receive the RREQ. The last-mile node in the route, as the nearest node to the sink, then replies the RREPs to the requested nodes. Subsequently, the RREP is acknowledged to the source node by the forwarders along the reverse route. In Figure 8, the possible connections are shown, among which a route has been selected based on the calculated λ scores.

#### 3.2.3. Selfish Node Management

**Figure 9:**
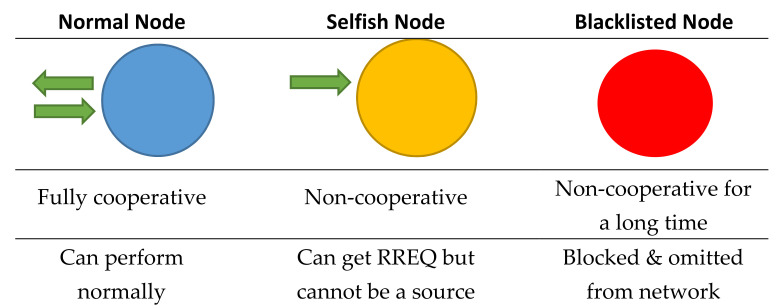
Stages for managing non-cooperative nodes.

It is possible that a node with the highest *λ* does not respond to an RREQ during *n* number of attempts. The source node then marks such a node as a selfish node and recalculates the value of *λ* by considering the remaining nodes. Moreover, the source node piggybacks the address of the selfish node with its RREQ to let the other nodes know about it. The nodes stop entertaining the selfish nodes once they get their information. However, selfish nodes can still be requested for the route. If the selfish node entertains an RREQ, then the source node announces it as a normal node again. In case a node does not respond to an *s* number of RREQs, it is then broadcasted as a blacklisted node. The network nodes omit the blacklisted nodes from their connected nodes’ lists for a specified period of time *t*. The features and capabilities of nodes in terms of their cooperation levels are indicated with blue, yellow, and red in Figure 9.

#### 3.2.4. Information Exchange

Upon each data transmission, the involved nodes consume their energies. These nodes update their λ values according to their knowledge. Using the DSR protocol [43] as a base protocol, each involved node can get the list of all the relay nodes. They update the values of the involved nodes in their routing tables. However, to avoid further energy consumption, these nodes do not broadcast their updated values. The nodes update their routing tables with approximate values by predicting the energy consumption and the number of transmitted packets.

**Figure 10:**
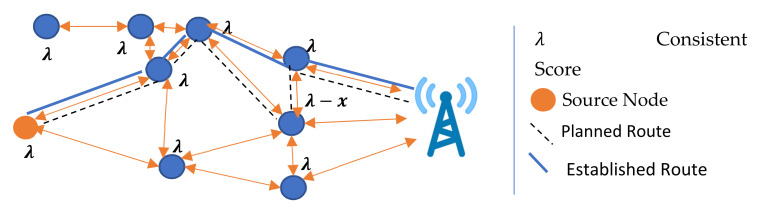
Updating statistics through RREP.

If a node has not received any updated information about other nodes in a possible route, it selectively sends a route confirmation to a preferred forwarder according to its knowledge. The source node does not proactively update the values, the λ of the relay node, which may be changed due to previous data transmissions. Therefore, the relay node selectively forwards the RREQ of the source node to its sibling neighbor node with the highest λ as shown in Figure 10.

The node also sends additional information with the RREQ that reflects the values of the nodes involved in previous data transmissions. The nodes in such a case search out the route by contacting upper-hop nodes with an RREQ along with the additional information. In this mechanism, the fundamental DSR-based technique of the RREQ broadcast is not used. The source and relay nodes selectively send the RREQ to the preferred nodes in the upper or similar hops.

Sometimes the source node does not receive acknowledgment of a selfish node after several attempts. In such a case, the source node either broadcasts the RREQ to its neighbors or selects another node according to its λ score. Each entry in the routing table is associated with a time stamp. This decision is made according to the time stamp attached to the next node’s stored λ score in the routing table. Each entry in the routing table is updated along with the current time.

## 4. Simulation Results

The proposed work was simulated using MATLAB 2018a. The list of simulation parameters is given in Table 1. The associations of the ***λ*** values in the first experiment are shown with the targeted parameters.

**Figure 11:**
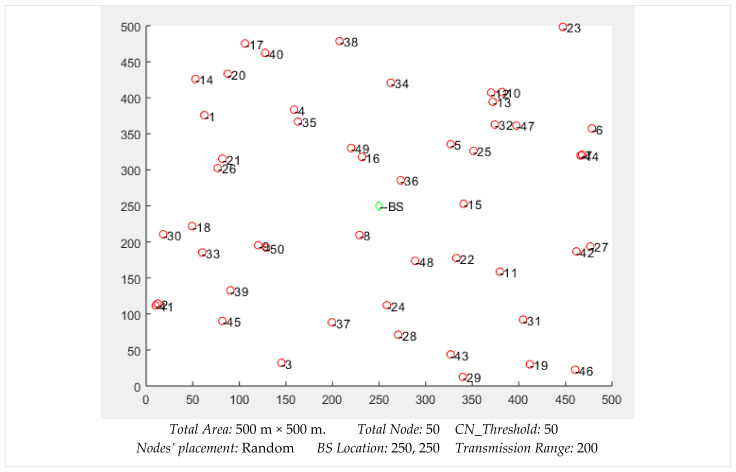
Placement of 100 nodes in an area of 500 m × 500 m.

The placements of 100 nodes can be seen in Figure 11. All the nodes were distributed evenly, while the location of the sink, labeled as BS, was kept at the center of the simulation space. We observed that the nodes were densely deployed in some places, while some nodes had low neighbor density according to their location. The nodes’ placements highly affected the network throughput, especially the availability and lifetime of the routes.

**Figure 12:**
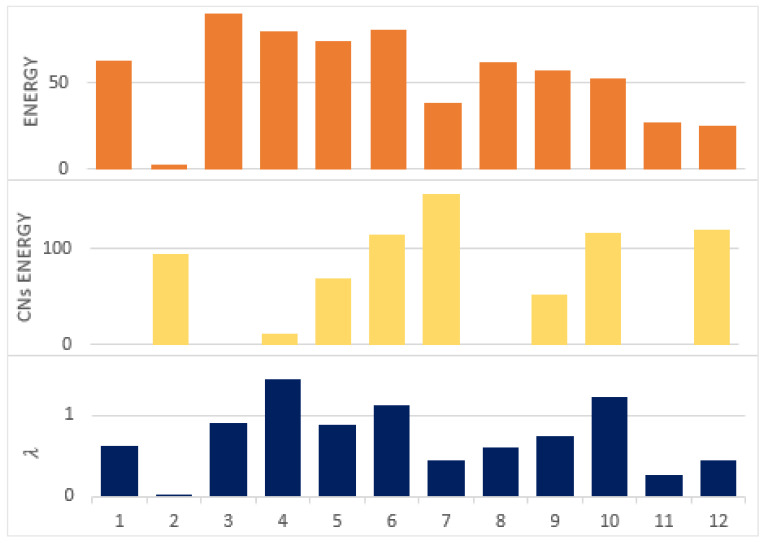
λ values according to nodes’ energies and CNs’ energies.

In the experiment scenario of Figure 11, the λ values for a sample of 12 nodes were derived, as shown in Table 2. The node that had an ID = 10 with a higher number of CNs had a comparatively higher λ value. This is because nodes with multiple CNs will get more route requests than others. Since their elimination from the network will not affect much due to the presence of multiple CNs, such nodes will be frequently utilized. Moreover, a node that had multiple CNs but less energy compared to its CNs had a lower value. For such cases, the node ID = 6 can be compared with the node ID = 7. Both had an equal number of CNs but different levels of energy. Therefore, different λ values were assigned to ID = 6 and ID = 7. Figure 12 shows a clear relationship among the nodes’ energies, their CNs’ energies, and the computed values of λ.

This work was further compared with some other protocols such as the PDORP, PRRP, DSR, and LEACH [44]. Experiments were performed to check the energy consumption, network life, throughput, and delays.

**Figure 13:**
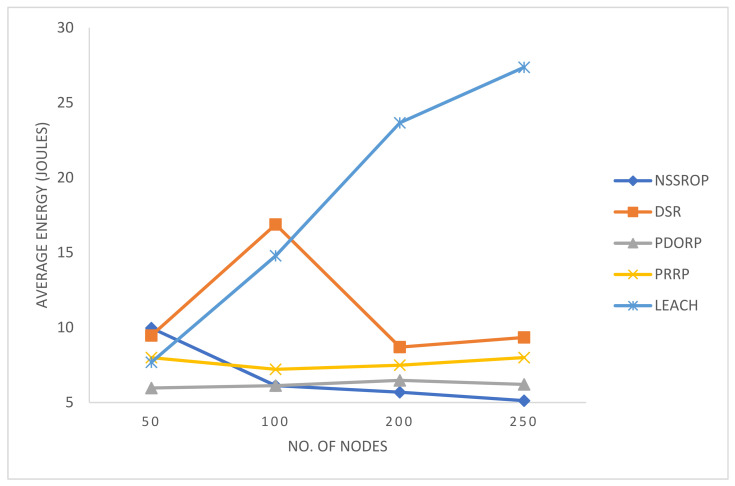
Average energy consumption by 5 protocols with a varying number of nodes.

Figure 13 shows the comparative results for energy consumption in all the experimented protocols. LEACH is not very sophisticated compared to modern protocols, but it is very famous for creating a baseline for other protocols. Many studies adopt LEACH as a base protocol for designing and comparing their work. In our results, its performance decreased with the increased number of nodes. The PDORP and PRRP obtained consistent results in terms of energy efficiency. The authors who developed the PDORP claimed to obtain encouraging results by using the genetic algorithm with a modified DSR. The PRRP was better than the DSR and LEACH but could not compete with the others. The key reason for this is the incorporation of a typical GPS in the nodes. The proposed mechanism did not perform well with a lower number of nodes, such as 50, because the NSSROP operates on the scores that are based on the nodes’ neighborhoods and densities. In the case of a smaller number of nodes, there were fewer or no CNs and identical CNs. Therefore, the proposed mechanism failed to obtain distinctive features from its key parameters with a lower number of nodes. However, a WSN-based IoT network mostly consists of a large number of devices. In such a dense network, therefore, the NSSROP worked much better than the other protocols; the NSSROP outperformed the other protocols with a number of nodes greater than 100.

**Figure 14:**
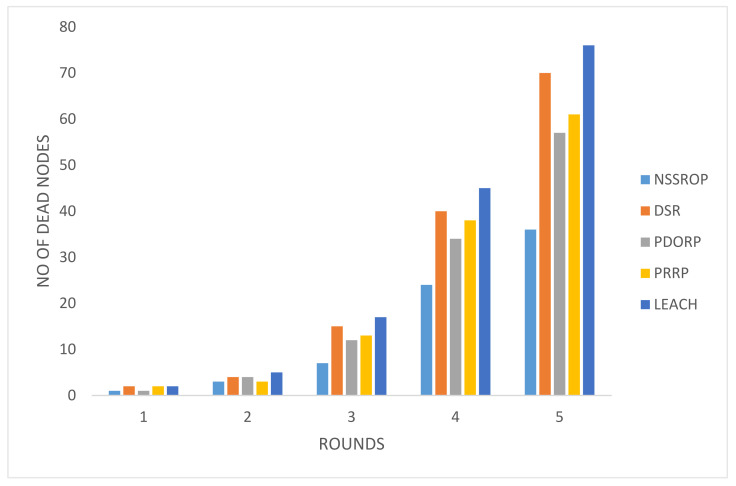
Number of dead nodes with different pause times.

The results for the ratio of dead nodes against five pause times can be seen in Figure 14. These results were reflected by the previous experiment on energy consumption. The NSSROP also outperformed in this test. Due to the equal load balancing, our protocol allowed all the nodes to equally participate in the network. Moreover, the blacklisting mechanism was also effective by not letting other nodes waste their energies on contacting them.

**Figure 15:**
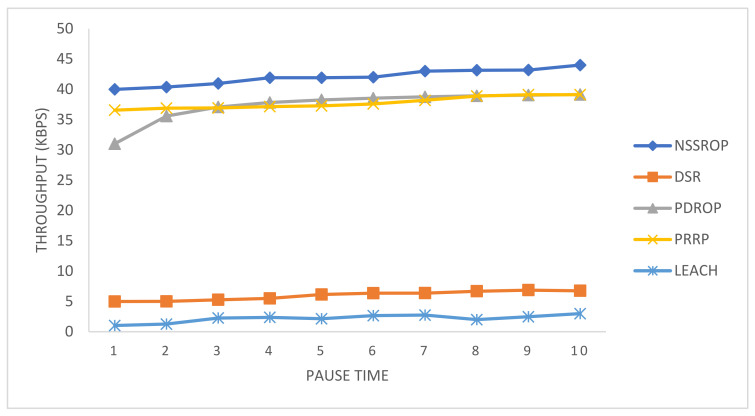
Throughput (packets per second) at time intervals.

Figure 15 shows the comparative results for the throughput of the experimented protocols. The PDROP and PRRP somehow obtained similar results. The DSR and LEACH had very poor throughput in the experiment. The PRRP has a mechanism that operates on fixed-sized grids and does not rely on the time duration; therefore, it had a relatively consistent level of throughput. The PDORP initially took time to implement its hybrid mechanism of a genetic algorithm and bacterial foraging optimization. As shown in the figure, the NSSRP achieved a comparatively higher throughput than the others. The main reason for this is the implementation of modified control packets, that is, RREQ, RREP, and OLSR-based topology control messages. With these modifications and incorporation of scoring, the packet drops decreased, and the exchange of data increased, causing a higher throughput.

**Figure 16:**
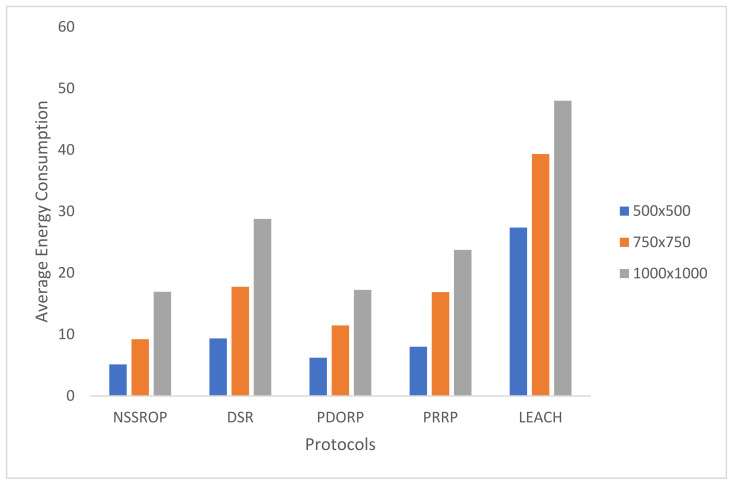
Average energy consumption with varying area size.

An experiment to check the impact of the density of nodes was carried out by varying the area up to 1000 m^2^ with a set of 250 nodes. The results in Figure 16 show that the performance of all the protocols degraded with an increased area size. This is because the scattered nodes may not have been able to gain multiple routes due to longer inter-node distances. The NSSROP obtained almost similar results to the PDORP for 1000 m^2^. However, due to the utilization of the density aspect, the performance of the NSSROP was much better for 500 m^2^ against the PDORP. Such a higher density increased the number of CNs and subsequently caused equal load distribution among the nodes and better selection among multiple routes.

**Figure 17:**
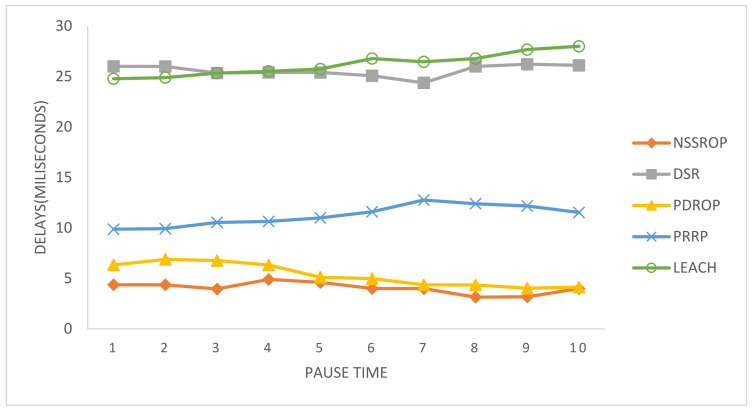
Average end-to-end delays with time pauses.

In Figure 17, the end-to-end delays are shown. The DSR and LEACH had the worst results due to their outdated routing procedures. DSR uses the typical route discovery mechanism that has a drawback of higher delays. The PRRP had moderate values for this experiment. The PDORP and NSSROP had almost similar results for end-to-end delays. The main reason for this is the incorporation of modified routing tables and the exchange of periodic topology control messages along with on-demand route discoveries. Moreover, the selection of appropriate routes also had a significant impact on the end-to-end delays.

## 5. Conclusions and Future Work

In this work, we proposed a new routing protocol, the NSSROP, which balances the load efficiently among the nodes in a WSN-based IoT environment. We implemented the NSSROP on top of two base protocols, the DSR and OLSR, with the novel scoring mechanism for path selection. Each node is scored considering its energy and CNs to indicate the nodes’ densities. In addition, the blacklisting mechanism is defined to deal with non-cooperating nodes in the WSN. In the experiment, the NSSROP showed outstanding results in terms of average energy consumption, throughput, and end-to-end delay.

This work can be further expanded by incorporating game theory and using clusters or groups in the network. A Stackelberg or evolutionary game can be incorporated into the mechanism for cluster formation and cluster head selection processes. Moreover, the same mechanism can be modified to design a scheme for the development of trusted routes while considering selfish nodes in the network. Many trust management systems have been proposed. The existing trust management systems, focusing on trust development from node to node, can be extended to the trust development for entire routes in the network.

## Figures and Tables

**Table 1:** List of simulation parameters.

Parameter	Value
Number of nodes	50 to 250
Area	500 × 500, 750 × 750, and 1000 × 1000 m^2^
Max propagation	100 meters
Max RSSI	100
CN threshold	50
Distance for identical nodes	25
Location of sink	250, 250
Energy max	100 J
Base protocols	DSR, OLSR
Node distribution	Random
Rx power	0.6 W
Tx power	0.6 W
Movement trace	Off
Comparisons	LEACH, DSR, PDORP, PRRP, NSSROP

**Table 2:** λ scores influenced by other parameters.

ID	Energy	CNs	CNs Energy	CN Energy Sum	*λ*
1	62.7347	0	0	0	0.6273
2	02.1650	41	94.5579	94.5579	0.0223
3	91.0570	0	0	0	0.9106
4	80.0559	35	10.6942	10.6942	1.4464
5	74.5847	25	68.3839	68.3839	0.8859
6	81.3113	7 44	38.3306 76.6831	115.0137	1.1345
7	38.3306	6 44	81.3113 76.6831	157.9944	0.4457
8	61.7279	0	0	0	0.6173
9	57.5495	50	52.7847	52.7847	0.7533
10	53.0052	12 13 32 47	24.8629 45.1639 21.7802 24.4165	116.2235	1.2257
11	27.5070	0	0	0	0.2751
12	24.8629	10 13 32	53.0052 45.1639 21.7802	119.9493	0.4522
